# Generalized Anxiety Disorder and Its Relationship with Dental Anxiety among Pregnant Women in Dammam, Saudi Arabia

**DOI:** 10.1155/2022/1578498

**Published:** 2022-02-22

**Authors:** Muhammad Ashraf Nazir, Mishali AlSharief, Asim Al-Ansari, Ahmed El Akel, Fai AlBishi, Shahd Khan, Gadah Alotaibi, Soha AlRatroot

**Affiliations:** ^1^Department of Preventive Dental Sciences, College of Dentistry, Imam Abdulrahman Bin Faisal University, Saudi Arabia P. O. Box 1982, Dammam 31441; ^2^College of Dentistry, Imam Abdulrahman Bin Faisal University, Saudi Arabia P. O. Box 1982, Dammam 31441

## Abstract

**Objective:**

To assess the relationship between generalized anxiety disorder (GAD), dental anxiety (DA), and other factors among pregnant women.

**Methods:**

The Generalized Anxiety Disorder (GAD-7) scale and the Modified Dental Anxiety Scale (MDAS) were used to evaluate GAD and DA, respectively. Hard copies of self-administered questionnaires were distributed among 780 pregnant women attending hospitals/health centers in Dammam, Saudi Arabia.

**Results:**

About 31.7% of the participants demonstrated minimal anxiety, 37.9% mild anxiety, 19.7% moderate anxiety, and 10.6% severe anxiety. The mean GAD score of the sample was 7.53 ± 5.16 which differed significantly among women in the first (7.74 ± 5.47), second (6.82 ± 4.64), and third trimesters (8.13 ± 5.37) (*P*=0.029). Pregnant women who performed routine dental visits demonstrated lower GAD (6.98 ± 5.23) than those who visited dentists for consultation, pain, or treatment (7.58 ± 5.07). The mean GAD score was significantly higher among participants with dental pain or discomfort during the last 12 months (8.12 ± 5.05) than among those without pain or discomfort (7.02 ± 5.2) (*P* 0.003). Similarly, the participants with DA showed a significantly higher mean GAD score (7.69 ± 5.17) than those without DA (5.93 ± 4.71) (*P* 0.006). Multivariate linear regression showed that GAD significantly correlated with DA (*B* = 0.225, *P* < 0.001).

**Conclusion:**

In the present study, GAD was common among pregnant women which significantly correlated with DA. Pregnant women with DA and dental pain demonstrated high GAD. Preventive and therapeutic measures should be taken to reduce GAD and DA during pregnancy.

## 1. Introduction

Generalized anxiety disorder (GAD) was first described as a chronic free-floating anxiety in 1959 [1]. It was recognized as a psychiatric condition in the third edition of the Diagnostic and Statistical Manual of Mental Disorders (DSM-III) in 1980, and modifications to its classification continued in the DSM-IV-TR [2]. GAD is characterized by excessive and uncontrollable worry and anxiety about day-to-day activities or situations for at least 6 months, and this causes distress leading to impairment of work, finances, health, etc. The condition is often associated with fatigue, restlessness, insomnia, muscle tension, poor concentration, gastrointestinal symptoms, and chronic headaches [2].

The lifetime prevalence of GAD was 7.7% in adult females and 4.6% in males in the United States [3]. The prevalence of GAD is higher in pregnant women (9.5%) [4] than in the general population (1.2%–6.4%) [5]. Pregnant women with anxiety disorders are three times more likely to have severe postpartum depression [6]. A systematic review of cohort studies reported a statistically significant association between anxiety among pregnant mothers and an increased risk of preterm birth (relative risk = 1.50) and low-weight birth (relative risk = 1.76) [7]. Pregnant women under the age of 25 years and those with a medical condition are at an increased risk of anxiety disorder [8].

A systematic review and meta-analysis by Kisely et al. indicated associations between psychiatric disorders and greater caries experience and increased tooth loss [9]. Individuals with mental disorders are more likely to report unmet dental needs, [10] demonstrate poor oral hygiene due to low interest in self-care, dryness of the mouth due to antidepressants, [11] periodontal disease (bleeding on probing, calculus, and periodontal pockets), [12] and temporomandibular disorders [13]. The literature also points out a bidirectional relationship between mental health and oral health. On one hand, the provision of dental treatment can cause anxiety and phobia, and the perception of dental pain may also increase anxiety and depression. On the other hand, many psychiatric problems increase the risk of oral diseases. For instance, patients with mental disorders are 2.7 times more likely to lose their teeth compared with individuals without mental disorders [14].

Dental anxiety (DA) is defined as “the response to a stressful stimulus that is specific to the dental context and its recognition should be established by reference to its origin” [15]. DA is common in women and it is significantly higher in women than in men [16, 17]. According to the Adult Dental Health Survey (2009), 16.7% of women demonstrated greater DA in the UK [18]. Patients with DA are known to avoid dental treatment and have compromised oral health and poor oral health-related quality of life [18, 19]. It is also confirmed that DA in women is associated with increased caries experience and poor oral health status [20].

The symptoms of GAD in pregnant women may be ignored/misdiagnosed by healthcare workers because such symptoms are related to pregnancy itself. However, early detection, effective diagnosis, and management of GAD are crucial for the provision of high-quality care. It is also important to understand GAD and related sociodemographic and dental factors among pregnant women. There is a lack of information in the literature about GAD during pregnancy and its association with DA, dental pain or problems, and routine dental attendance. Medical and dental professionals may utilize study findings to improve the general and oral health of pregnant women. The study aimed to evaluate GAD and factors associated with it during pregnancy in Greater Dammam, Saudi Arabia.

## 2. Materials and Methods

### 2.1. Study Settings and Design

This cross-sectional study was conducted at different hospitals and health centers in Greater Dammam, Saudi Arabia. Greater Dammam is a large metropolitan area that has a population of 1.75 million people and consists of the triplet cities of Dammam, Khobar, and Dhahran in the Eastern Province.

### 2.2. Population

Recruitment of participants for the study was restricted to pregnant women visiting prenatal clinics at hospitals and health centers in Greater Dammam. The study population encompassed pregnant women of any age and those who attended prenatal clinics during the period from June to August 2019. The assumptions of 95% confidence level, 3% margin of error, 50% response distribution, and population size (*N* ≈  20,000) were considered for sample size calculation, and an estimated sample of 1014 pregnant women was used in the study. Enrollment in the study was based on a convenience sampling method. Pregnant women who voluntarily participated and signed a letter of informed consent were enrolled in the study. The subjects with cognitive inability to comprehend items of the questionnaire and the participants who provided incomplete questionnaires or illegible responses were excluded from the study.

### 2.3. Instruments

The self-administered questionnaire included the Generalized Anxiety Disorder (GAD-7) Scale, the Modified Dental Anxiety Scale (MDAS), and demographic information. The severity of GAD symptoms was assessed by using a 7-item scale of a generalized anxiety disorder (GAD-7) [21]. The GAD-7 is a clinically useful tool (61.3%–73.3% of sensitivity and 67.3%–72.7% of specificity) for the detection of GAD during pregnancy [22, 23]. The seven items of the GAD-7 are “nervousness,” “inability to stop worrying,” “excessive worry,” “restlessness,” “difficulty in relaxing,” “easy irritation,” and “fear of something awful happening.” Participants rate their anxiety experiences over the past 2 weeks by responding to the items on the scale. Each item of the GAD-7 scale has a 4-point Likert scale (“not at all” (0), “several days” (1), “more than half the days” (2), and “nearly every day” (3)). The total score of GAD-7 ranges from 0 to 21 and it is divided into four categories: minimal anxiety (0–4), mild anxiety (5–9), moderate anxiety (10–14), and severe anxiety (15–21) [24]. The factorial validity, concurrent validity, and reliability (Cronbach's alpha = 0.89) of the GAD-7 scale have been confirmed in previous studies [21, 23].

The study used the MDAS scale to evaluate dental anxiety (DA) among pregnant women [25]. Compared with other instruments used for the evaluation of DA, MDAS has the advantage of quick administration and detection of patients with DA in epidemiological studies. MDAS consists of five items on a 5-point Likert scale (from “not anxious” (1) to “extremely anxious” (5)). The score of the MDAS scale ranges from 5 to 25. Previous studies confirmed the reliability and validity of the MDAS scale [26, 27]. The participants also provided information about age, nationality, educational attainment, income level, medical history, the experience of pain or discomfort in teeth or mouth during the last 12 months, dental attendance, current month of pregnancy, and the number of previous pregnancies. Arabic versions of GAD-7 and MDAS were used in the study [26, 28]. Both English and Arabic language questionnaires were used among the non-Arab and Arab study populations, respectively. Pilot testing of the questionnaire was performed before its final administration.

### 2.4. Procedures

After obtaining permission from hospitals and health centers, self-administered questionnaires were administered among pregnant women in the waiting areas of prenatal clinics. The female researchers visited hospitals/health centers for data collection. They provided study details to the participants and addressed their queries regarding the research during questionnaire administration.

### 2.5. Ethical Considerations

The present investigation is part of the research protocol (EA: 2019040) that was reviewed, approved, and registered by the Scientific Research Unit (ethics and research committee) at the College of Dentistry, Imam Abdulrahman Bin Faisal University, Dammam. The study adhered to ethical principles regarding research on human beings in the Declaration of Helsinki. The participants voluntarily participated in the study and provided written informed consent. The administration of an anonymous survey maintained the privacy and confidentiality of the participants.

### 2.6. Data Management and Statistical Analysis

Sociodemographic data and scores of the GAD and DA scales were entered into a Microsoft Excel (2010) database. Statistical analysis was performed by using IBM SPSS version 25 (IBM Corp., Armonk, NY, USA). Frequencies and proportions were calculated for categorical variables and means, and standard deviations were calculated for continuous data. Normal distributions of GAD and DA were evaluated by using the Kolmogorov test. Accordingly, the Mann–Whitney *Ut*-test and the Kruskal–Wallis test were performed to compare scores of GAD in two or more than two categories of participants, respectively. The correlation between GAD and DA was assessed in univariate and multivariate linear regression analyses. A *P*value of less than 0.05 was considered statistically significant.

## 3. Results

The participants returned 825 completed questionnaires; however, 45 questionnaires were excluded from the analysis because of incomplete responses about generalized anxiety disorder (*N* = 780). More than half of the sample consisted of Saudi women (64.1%) and had a university education (65.8%). Dental anxiety (mild-extreme) was found in 90.9% of the participants. The mean GAD score was significantly higher among participants with dental pain or discomfort during the last 12 months (8.12 ± 5.05) than among those without pain or discomfort (7.02 ± 5.2) (*P*=0.003). Similarly, the participants with DA showed a significantly higher mean GAD score (7.69 ± 5.17) than those with no DA (5.93 ± 4.71) (*P*=0.006). There were significant differences in mean GAD scores among participants in the first (7.74 ± 5.47), second (6.82 ± 4.64), and third trimesters (8.13 ± 5.37) (*P*=0.029). Pregnant women who performed routine dental visits demonstrated lower GAD (6.98 ± 5.23) than those who visited dentists for consultation, pain, or treatment (7.58 ± 5.07), although the difference was not statistically significant. The study found no significant differences in the mean scores of GAD with regards to nationality, education, monthly family income, number of pregnancies (Table 1).

The mean GAD score of the sample was 7.53 ± 5.16. According to GAD questionnaire items, “becoming easily annoyed or irritable” secured the highest mean score (1.31 ± 1.02) and this was followed by “feeling afraid as if something awful might happen” (1.17 ± 1.05). Becoming easily annoyed or irritable nearly every day was experienced by 20.9% of participants. On the other hand, “not being able to stop or control worrying” had the lowest mean score (0.85 ± 0.92) (Table 2).

**Figure 1:**
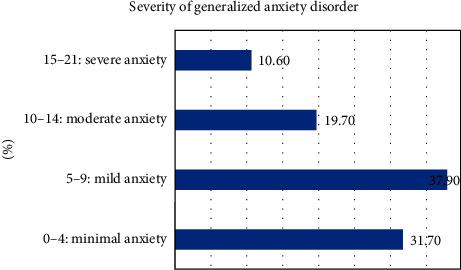
Distribution of the severity of generalized anxiety disorder among pregnant women.

Almost 31.7% of participants had minimal anxiety, 37.9% mild anxiety, 19.7% moderate anxiety, and 10.6% severe anxiety (Figure 1).

Univariate analysis showed that DA was significantly correlated with GAD (rho 0.240, *P* ≤ 0.001). No correlation was found between GAD and age, education, monthly income, current month of pregnancy, and the number of previous pregnancies. In multivariate analysis, a significant correlation remained between GAD and DA (*B* = 0.225, *P* < 0.001) independent of other factors (Table 3).

## 4. Discussion

Although dental treatment can be safely provided during pregnancy, the second trimester is considered safer than the first and third trimesters. In the first trimester, dental treatment that involves the administration of local anesthesia or medications may negatively affect the fetus during organogenesis. The supine position of pregnant women in the dental chair may lead to hypotension, syncope, and reduced uteroplacental perfusion during the third trimester. Routine dental care can be provided in the third trimester; however, the difficulty of sitting in the dental chair during the third trimester may increase discomfort levels and may hinder the provision of dental treatment. The risk to the fetus is low in the second trimester because organogenesis is complete, and dental treatment can be provided safely [29]. A previous study from Dammam showed that pregnant women more commonly visited the dentist in the second trimester (40.7%) than in the first trimester (25.4%) and third trimester (33.9%) (*P*=0.049) [30]. Interestingly, the present study found significant differences in the GAD scores in three trimesters with a lower score in the second than in the first and third trimesters. Since there is a significant relationship between GAD and DA in the present study, the low occurrence of GAD in the second trimester should be considered by medical professionals for patient referral to a dentist for effective patient management and improved dental care.

A study of Nigerian pregnant women showed that the presence of medical conditions was significantly associated with 3.6 times higher odds of having an anxiety disorder [8]. This finding seems in line with our study results, where significantly higher GAD was found in pregnant women with a medical condition versus those without a medical condition. The literature concerning the effects of GAD and DA on access to oral care and patient management during pregnancy is limited. It is important to understand the clinical implications of this relationship of GAD with medical conditions. Besides, the significant correlation of GAD with DA should be kept in mind when evaluating the association of GAD with medical conditions. Therefore, healthcare providers should consider the specific evaluation and treatment of GAD and DA during pregnancy.

There is an increased risk of periodontal disease and dental caries due to physiological hormonal changes and poor dietary and oral hygiene behaviors during pregnancy which can lead to dental pain or discomfort [31]. It is documented that depressive and/or anxiety disorders are associated with pain intensity and disability related to pain, musculoskeletal pain, cardiorespiratory pain, and gastrointestinal pain [32]. The exacerbation of anxiety and depression due to dental pain is also indicated in the literature [14]. This might explain the reason behind significantly higher GAD among pregnant women with dental pain or discomfort in the present study. A healthy diet, appropriate oral hygiene practices including the use of fluorides, and routine dental care prevent dental problems and dental pain which may also minimize prenatal GAD.

Routine dental visits are important for the maintenance of good oral health of mothers and their children [33, 34]. However, there is reduced dental care utilization during pregnancy, and routine dental visits are even much lesser than visits for pain or treatment. For instance, a considerable proportion of pregnant women (52.6%) in Saudi Arabia avoid visiting the dentist, and only 13.7% attend a dental office for a routine dental checkup [30]. Low routine dental attendance is reported in previous studies of pregnant women in the United Arab Emirates (14.4%) [35], Greece (19.6%) [33], and the U.S. (26%) [34]. Our study found lower GAD in pregnant women who performed routine dental visits compared to those who visited dentists for consultation, pain, or dental treatment before and during pregnancy. Healthcare providers should consider the importance of low GAD in relation to routine dental attendance and ensure efficient patient flow to dentists for optimal oral health during pregnancy.

In the present study, the pregnant women with DA demonstrated significantly higher GAD than those without DA. Similarly, the study also showed a statistically significant correlation between GAD and DA in both univariate and multivariate linear regression analyses. It is known that dentally anxious patients are more vulnerable to anxiety disorders. A Canadian study by Locker et al. found a higher prevalence of psychological disorders among dentally anxious (55.0%) than nonanxious individuals (42.3%) and that psychological disorders were related to long-term DA [36]. A study by Yildirim et al. observed a significant relationship between DA and state-trait anxiety among patients attending a periodontology clinic in Turkey [37]. A significant association between high DA and high state-trait anxiety was also reported by Fuentes et al. in Brazil [38]. Lago-Méndez demonstrated significant positive correlations between state-trait anxiety and DA and dental fear among patients in Spain [39]. In a study of dentally anxious patients in Sweden, Hakeberg et al. found a significant association between DA and general anxiety [40]. In another study from Sweden, Hägglin et al. followed adult women for more than two decades to investigate the longitudinal course of DA in relation to mental health. The authors observed associations between high DA with psychiatric impairment and high general anxiety [41]. Nevertheless, a previous study reported no significant differences in DA in individuals with low and high general fear [42].

DA can aggravate patients' behavior in dental practice and may prolong or complicate dental treatment. Dental practitioners, therefore, can take appropriate measures to prevent or control DA if they are aware of a patient's predilection to DA. However, there is a lack of literature on DA and GAD in pregnant women, hence the present study has clinical implications for both the dental and medical professions regarding the care of pregnant women. Medical practitioners should consider the relationship of GAD with DA when referring to pregnant women for dental care. The evaluation of GAD and DA should be conducted for the effective management of pregnant women in dental practice.

The present study has certain limitations. The cross-sectional study is limited in establishing a temporal link between exposure and outcome/cause and effect relationship because exposure and outcome are measured at the same time. Therefore, associations/relationships identified in the cross-sectional study may be difficult to interpret. There was a significant correlation between GAD and DA; however, the study results cannot be used to infer that increased GAD actually resulted in greater DA. A longitudinal study may shed more light on the actual impact of GAD in increasing the risk of DA. Furthermore, external validity is limited. A large sample of pregnant women from Greater Dammam was included in the present study. However, the study results may not be generalized to pregnant women in other cities or rural areas of Saudi Arabia. The use of validated and reliable questionnaires provides robust study results; however, there is a possibility of under and overreporting in survey studies.

## 5. Conclusions

The study showed statistically significant differences in the three trimesters with the highest anxiety observed in the third trimester. There was a statistically significant correlation between GAD and DA. The participants with DA demonstrated significantly higher GAD than those without DA. Low GAD was found among participants who performed routine dental visits, and high GAD was related to dental pain or discomfort and medical condition. The study results call for interdisciplinary collaboration among health care providers to take preventive measures for the reduction of GAD and DA. Physicians should be aware of the distribution and consequences of GAD among pregnant women so that they can effectively manage them during treatment. They should understand the relationship between GAD and DA and dental attendance when referring pregnant women for dental care. Similarly, dental professionals should screen pregnant women for GAD and DA to ensure effective patient management and the provision of high-quality dental treatment.

## Figures and Tables

**Table 1:** Distribution of sociodemographic factors and their relationship with generalized anxiety disorder in pregnant women.

Study variables	*N* (%) (*N* = 780)	Generalized anxiety disorder score (mean ± SD)	*P*value
Nationality			0.938
Saudi	500 (64.1)	7.60 ± 5.28	
Non-Saudi	280 (35.9)	7.41 ± 4.93	
Number of previous pregnancies			0.375
First pregnancy	209 (26.8)	7.15 ± 4.95	
Second pregnancy	248 (31.8)	7.40 ± 5.37	
Third pregnancy	169 (21.7)	7.83 ± 4.97	
Four or more pregnancies	154 (19.7)	7.94 ± 5.29	
Level of education			0.526
No education	12 (1.5)	8.67 ± 5.91	
School education	255 (32.7)	7.84 ± 5.48	
University or higher level	513 (65.8)	7.35 ± 4.97	
Monthly family income			0.664
2,000–6,000 SAR.	315 (40.4)	7.75 ± 5.42	
6,000–12,000 SAR.	308 (39.5)	7.54 ± 5.08	
More than 12,000 SAR	157 (20.1)	7.09 ± 4.74	
Current month of pregnancy			0.029^*∗*^
1–3 months (first trimester)	202 (25.9)	7.74 ± 5.47	
4–6 months (second trimester)	295 (37.8)	6.82 ± 4.64	
7–9 months (third trimester)	283 (36.3)	8.13 ± 5.37	
Medical problems			0.050
Yes	112 (14.4)	8.35 ± 5.08	
No	668 (85.6)	7.4 ± 5.16	
Pain or discomfort in teeth or mouth during the last 12 months			<0.001^*∗*^
Yes	363 (46.5)	8.12 ± 5.05	
No	417 (53.5)	7.02 ± 5.2	
Dental anxiety			0.003^*∗*^
Yes	709 (90.9)	7.69 ± 5.17	
No	71 (9.1)	5.93 ± 4.71	
Dental attendance before pregnancy (*N* = 648)			0.396
Routine dental visits	73 (11.3)	7.11 ± 5.18	
Consultation/pain/treatment or follow-up	575 (88.7)	7.55 ± 5.09	
Dental attendance during pregnancy (*N* = 381)			0.295
Routine dental visits	51 (13.4)	6.98 ± 5.23	
Consultation/pain/treatment or follow-up	330 (86.6)	7.58 ± 5.07	

^*∗*^Statistically significant.

**Table 2:** Generalized anxiety disorder (GAD): distribution of items' responses of pregnant women.

GAD questionnaire items	Mean ± SD	Not at all sure	Several days	Over half the days	Nearly every day
Feeling nervous, anxious, or on edge	0.98 ± 0.93	260 (33.3)	361 (46.3)	72 (9.2)	87 (11.2)
Not being able to stop or control worrying	0.85 ± 0.92	328 (42.1)	317 (40.6)	62 (7.9)	73 (9.4)
Worrying too much about different things	1.16 ± 0.98	198 (25.4)	380 (48.7)	79 (10.1)	123 (15.8)
Trouble relaxing	1.12 ± 0.99	227 (29.1)	341 (43.7)	100 (12.8)	112 (14.4)
Being so restless that it is hard to sit still	0.94 ± 0.92	283 (36.3)	338 (43.3)	83 (10.6)	76 (9.7)
Becoming easily annoyed or irritable	1.31 ± 1.02	162 (20.8)	375 (48.1)	80 (10.3)	163 (20.9)
Feeling afraid as if something awful might happen	1.17 ± 1.05	226 (29)	346 (44.4)	61 (7.8)	147 (18.8)

**Table 3:** Univariate and multivariate linear regression models: correlation between GAD and DA and other covariates.

Study variables	Univariate analysis		Multivariate analysis	
	Spearman's rho	*P*value	Unstandardized coefficients (B)	*P*value
Dental anxiety	0.240	<0.001^*∗*^	0.225	<0.001^*∗*^
Age	0.010	0.783	−0.025	0.554
Level of education	−0.037	0.298	−0.335	0.367
Monthly family income	−0.028	0.435	−0.291	0.244
Number of previous pregnancies	0.060	0.093	0.285	0.166
Current month of pregnancy	0.040	0.263	0.342	0.140

^*∗*^Statistically significant.

## Data Availability

The SPSS data file of this study is available from the corresponding author upon request.
